# Supplementary material for: Multi-angular polarimetric remote sensing to pinpoint global aerosol absorption and direct radiative forcing
Source: Nat Commun. 2022 Dec 3;13:7459. doi: 10.1038/s41467-022-35147-y (PMC9718735; doi:10.1038/s41467-022-35147-y)
Supplement: Supplementary file 1 — Supplementary information [file 41467_2022_35147_MOESM1_ESM.pdf]

Supplementary information to "Multi-angular polarimetric remote sensing to pinpoint global aerosol absorption and direct radiative forcing"

Cheng Chen[1,2,*], Oleg Dubovik[1,*], Gregory L. Schuster[3], Mian Chin[4], Daven K. Henze[5], Tatyana Lapyonok[1], Zhengqiang Li[6], Yevgeny Derimian[1] and Ying Zhang[6]

[1] Univ. Lille, CNRS, UMR 8518 - LOA - Laboratoire d'Optique Atmosphérique, F-59000 Lille, France

[2] GRASP-SAS, Univ. Lille, Villeneuve d'Ascq, 59650, France

[3] NASA Langley Research Center, Hampton, Virginia, 23681, USA

[4] NASA Goddard Space Flight Center, Greenbelt, Maryland, 20771, USA

[5] Department of Mechanical Engineering, University of Colorado, Boulder, Colorado, 80309, USA

[6] Aerospace Information Research Institute, Chinese Academy of Sciences, Beijing, 100101, China

Corresponding authors: Cheng Chen (cheng.chen@grasp-sas.com) and Oleg Dubovik (oleg.dubovik@univ-lille.fr)

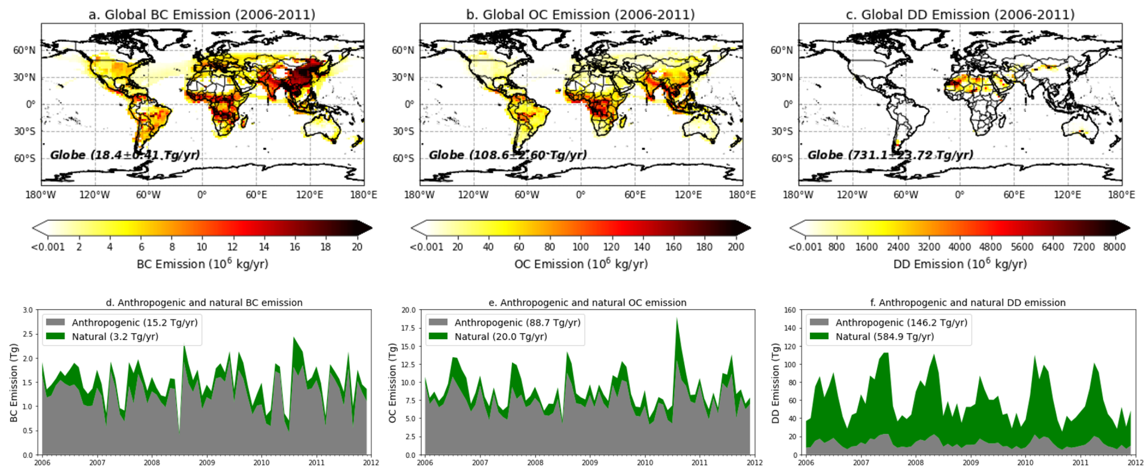

Supplementary Fig. 1. The MAP-constrained absorbing aerosol emission database (MACE) emission database of black carbon (BC), organic carbon (OC) and desert dust (DD). (a). Spatial distribution of annual mean BC emission from 2006 to 2011; **(b).** Spatial distribution of annual mean OC emission; **(c).** Spatial distribution of annual mean DD emission; **(d).** The monthly variation of anthropogenic and natural BC emission from 2006 to 2011; **(e).** The monthly variation of anthropogenic and natural OC emission; **(f).** The monthly variation of anthropogenic and natural DD emission.

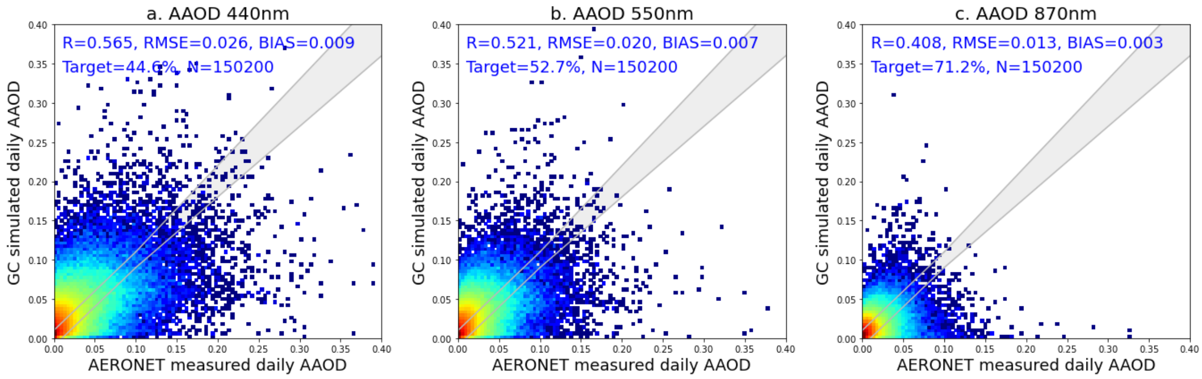

Supplementary Fig. 2. Evaluation of GEOS-Chem v11-01 simulation of daily spectral aerosol absorption optical depth (AAOD) using the MAP-constrained absorbing aerosol emission database (MACE) emission database with the Aerosol Robotic Network (AERONET) inversion products of AAOD at blue (440 nm), mid-visible (550 nm) and near infrared (870 nm) channels. (a) AAOD 440 nm; **(b)** AAOD 550 nm; **(c)** AAOD 870 nm. Statistical metrics for validation are presented including Pearson correlation coefficient (R), root mean square error (RMSE), bias (BIAS), fraction within target requirement (Target) and number of matchups (N).

Supplementary Table 1. Intercomparison of GEOS-Chem simulation of speciated aerosol optical depth (AOD) and direct radiative forcing (DRF) using a priori emission in 2010 (Heald et al.[1]) with this study using the MAP-constrained absorbing aerosol emission database (MACE) emission from 2006 to 2011. The speciated anthropogenic AOD are in the brackets.

	AOD at 550 nm		DRF (W/m^2)	
	A priori emission[2]	MAP-constrained emission[3]	A priori emission[2]	MAP-constrained emission[3]
BC[1]	0.0012 (0.0007)	0.008 (0.006)	0.078	0.33
OM[1]	0.0147 (0.0029)	0.033 (0.025)	-0.055	-0.05
DD[1]	0.021 (0.004)	0.018 (0.003)	-0.053	-0.026
SS[1]	0.032 (0)	0.029 (0)	0	0
SNA[1]	0.0226 (0.0151)	0.031 (0.019)	-0.331	-0.394
Total	0.092 (0.023)	0.119 (0.054)	-0.36	-0.14

[1] BC represents black carbon, OM for organic matter, DD for desert dust, SS for sea salt and SNA for Sulfate-Ammonium-Nitrate

[2] Values adopted from Heald et al.[1] based on the simulation in 2010

[3] MAP-constrained emission (MACE) from 2006 to 2011 used for simulations in this study

Supplementary References

1. Heald, C. L. et al. Contrasting the direct radiative effect and direct radiative forcing of aerosols. *Atmos. Chem. Phys.* **14**, 5513–5527 (2014).
